# Supplementary material for: DNA Damage Regulates the Functions of the RNA Binding Protein Sam68 through ATM-Dependent Phosphorylation
Source: Cancers (Basel). 2022 Aug 9;14(16):3847. doi: 10.3390/cancers14163847 (PMC9405969; doi:10.3390/cancers14163847)
Supplement: Supplementary file 1 [file cancers-14-03847-s001.zip › cancers-1727942-supplementary Figures.pdf]

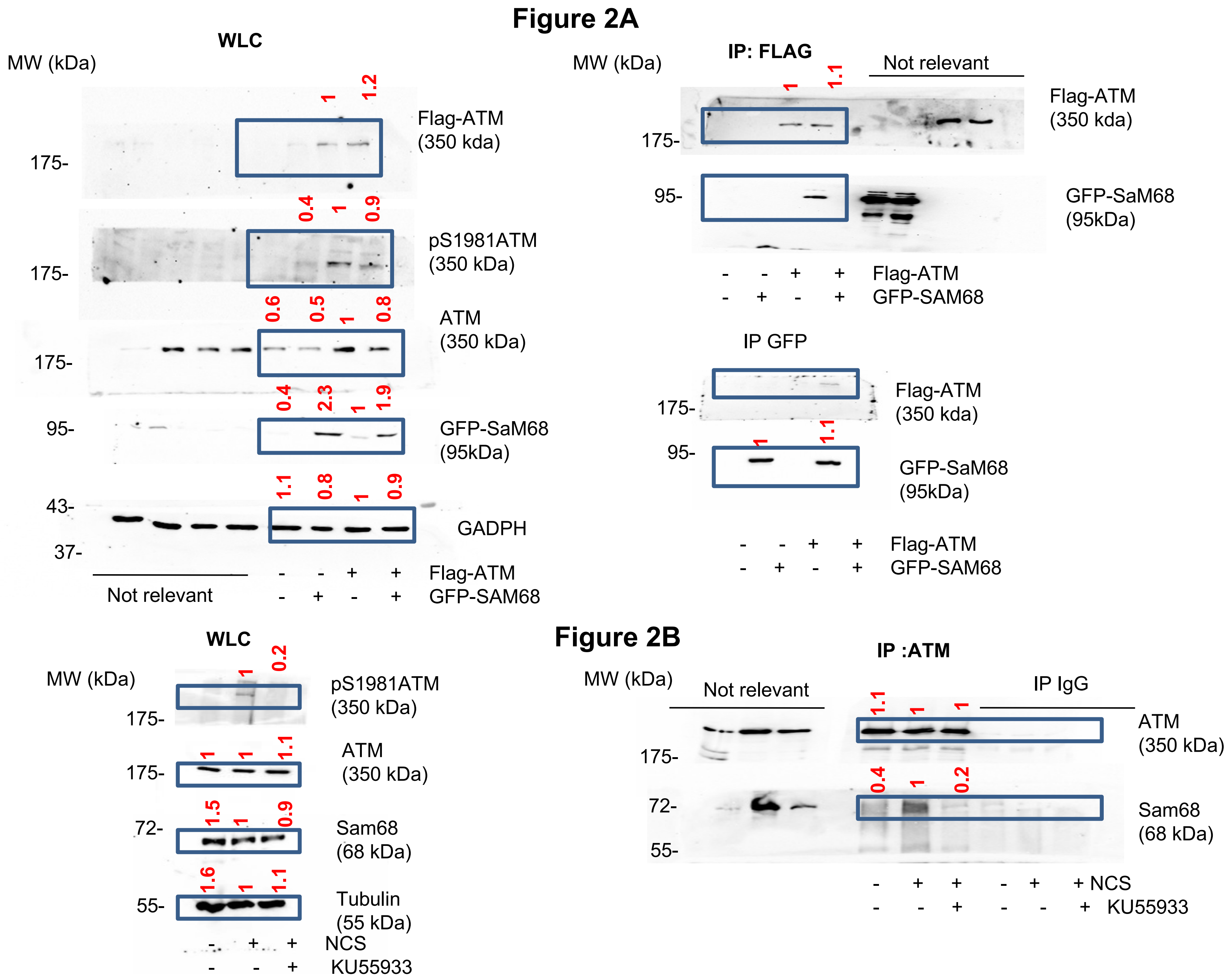


**Figure S1.** Uncropped Western blot of Figure 2 A and 2B and and normalized densitometry in red.

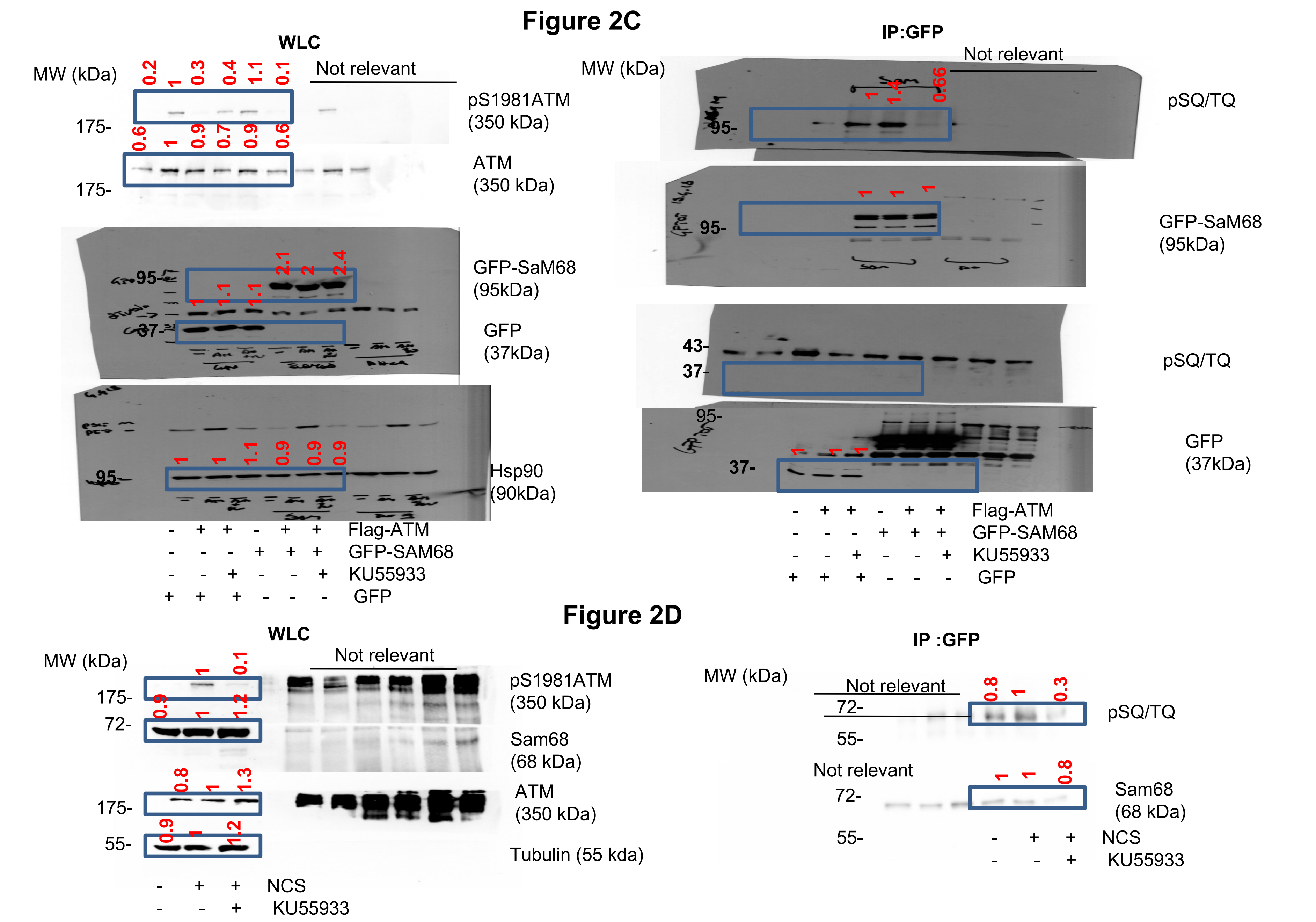


**Figure S1.** Uncropped Western blot of Figure 2 C and 2D and normalized densitometry in red.

## Figure 3B

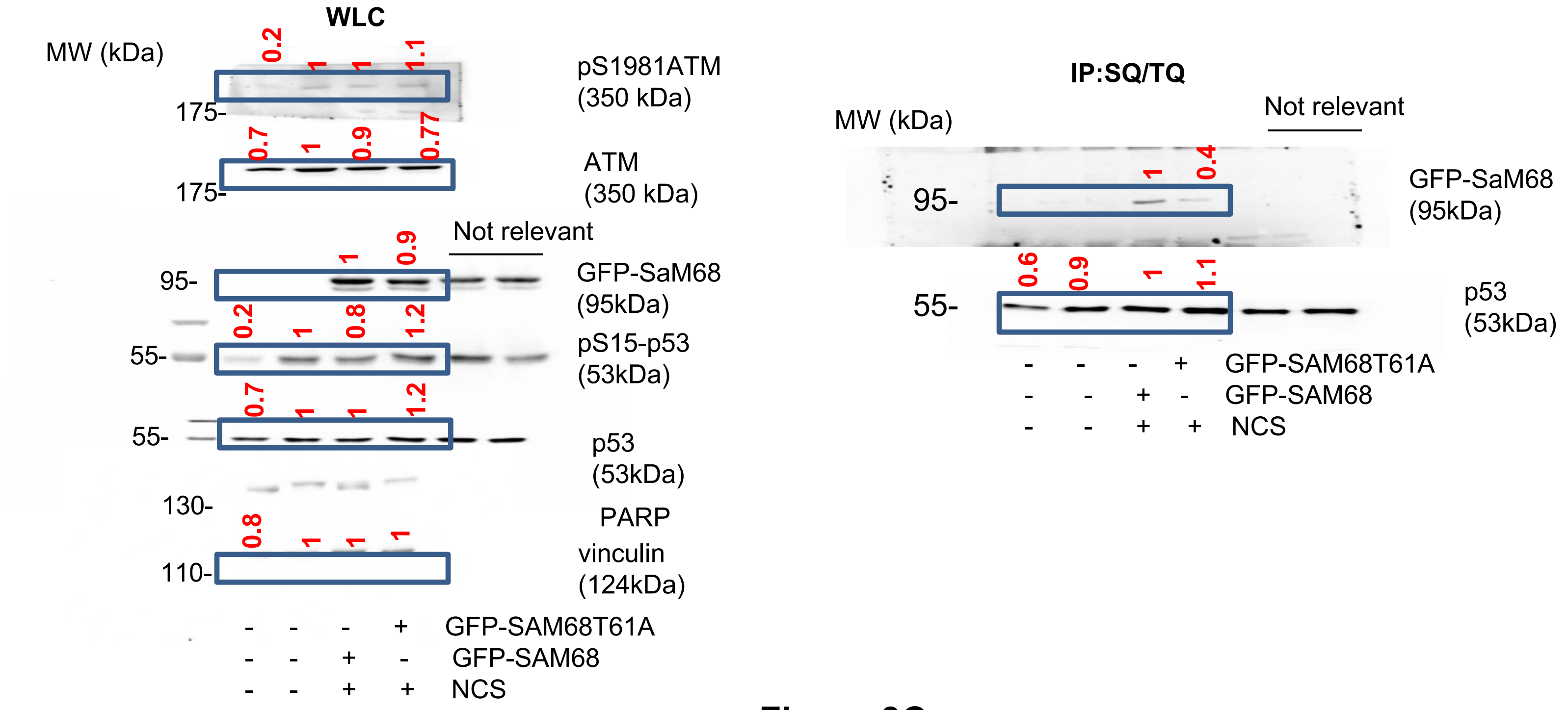


## Figure 3C

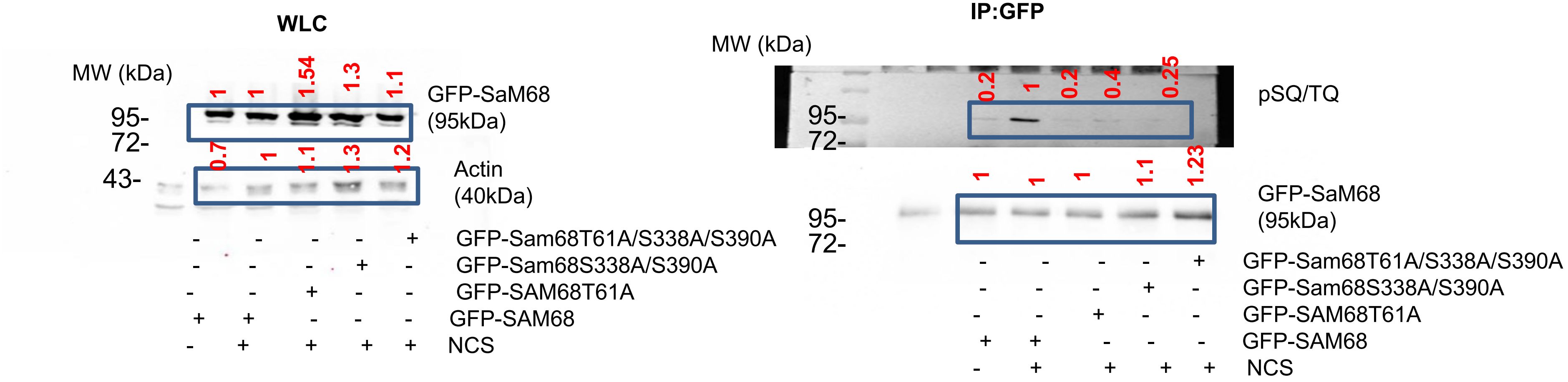


**Figure S1.** Uncropped Western blot of Figure 3 B and 3C and normalized densitometry in red.

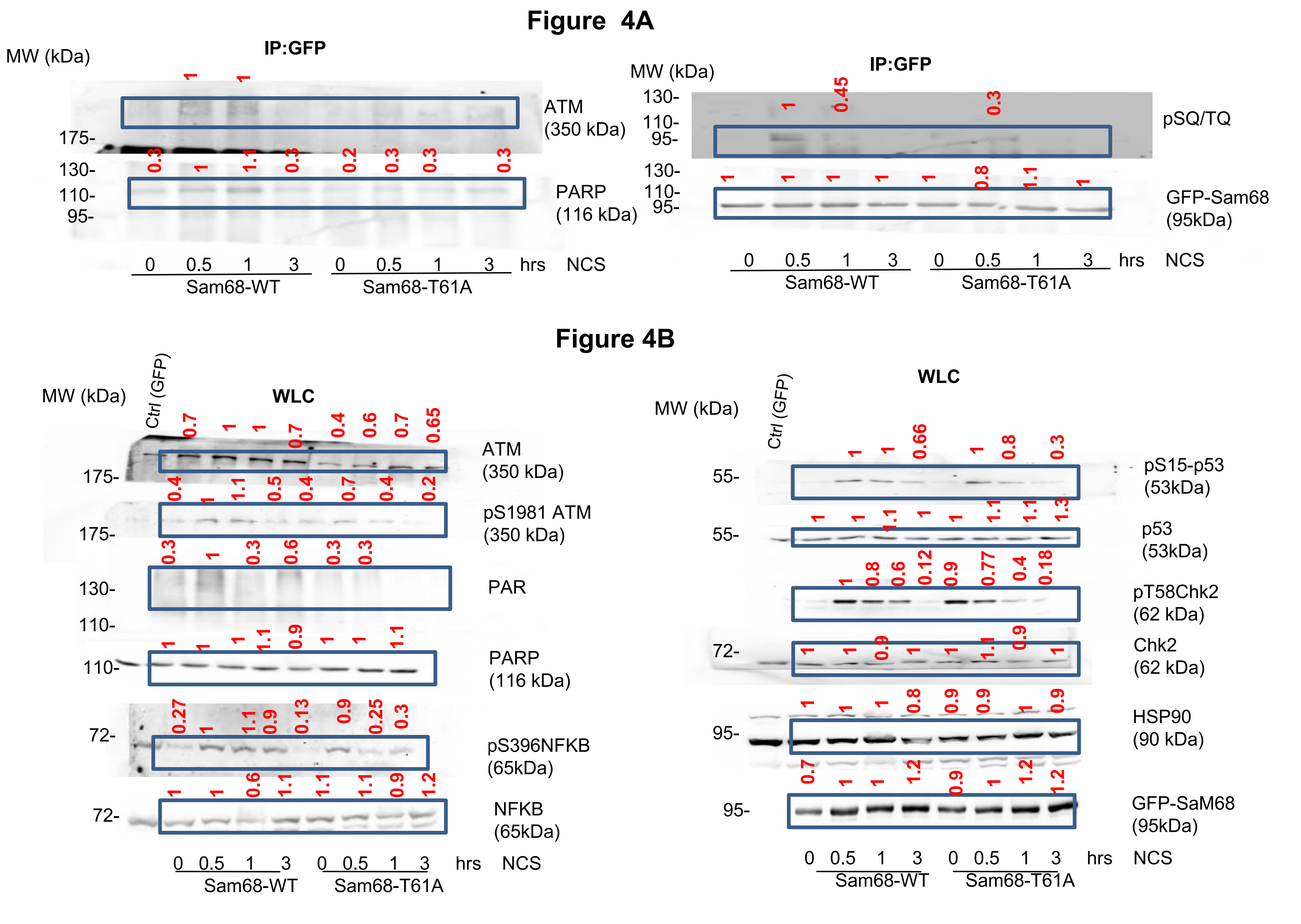


**Figure S1.** Uncropped Western blot of Figure 4A and 4B and normalized densitometry in red.

## Figure 4C

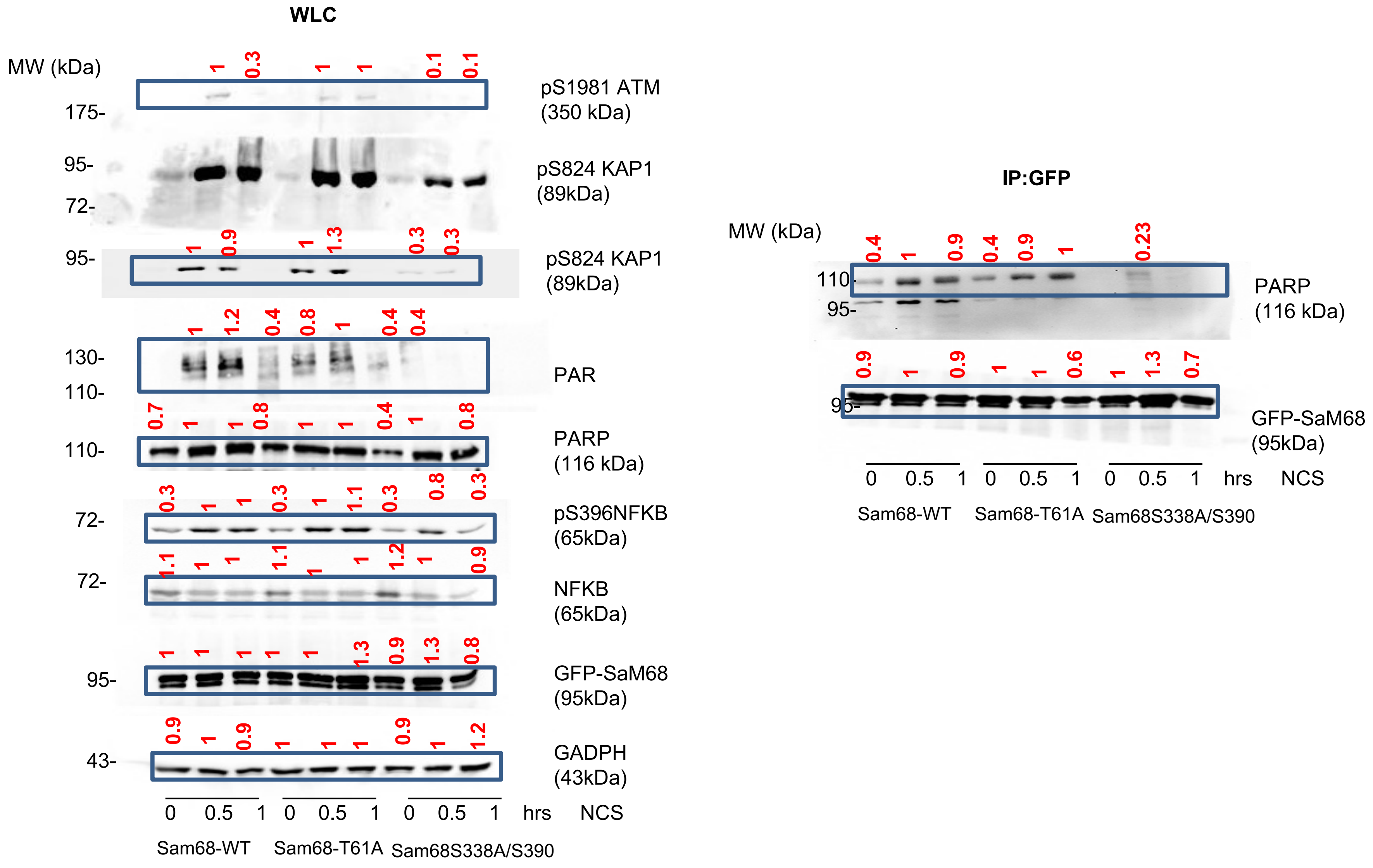


**Figure S1.** Uncropped Western blot of Figure 4C and normalized densitometry in red.

# Figure 5A

**PolyA pulldown**

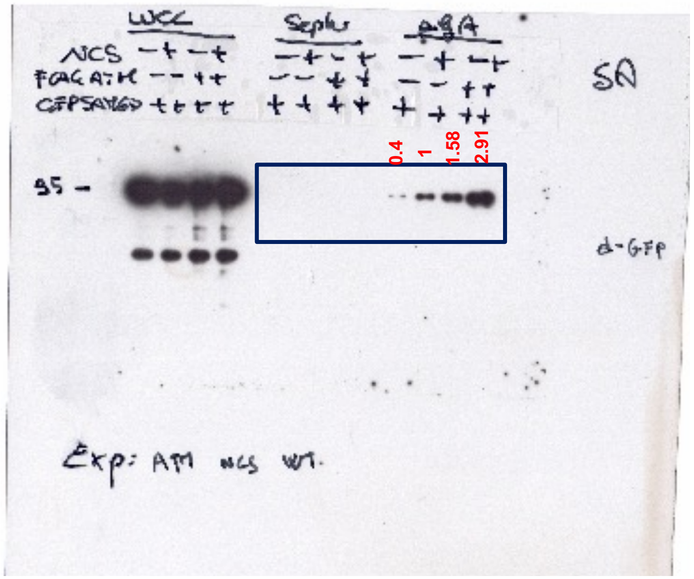


GFP-
Sam68
(95 kDa)

**WCL**

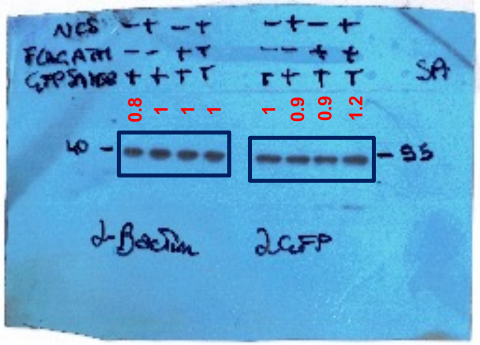


β-Actin
(40 kDa)

GFP-Sam68
(95 kDa)

**Figure S1.** Uncropped Western blot of Figure 5A and normalized densitometry in red.

# Figure 5B

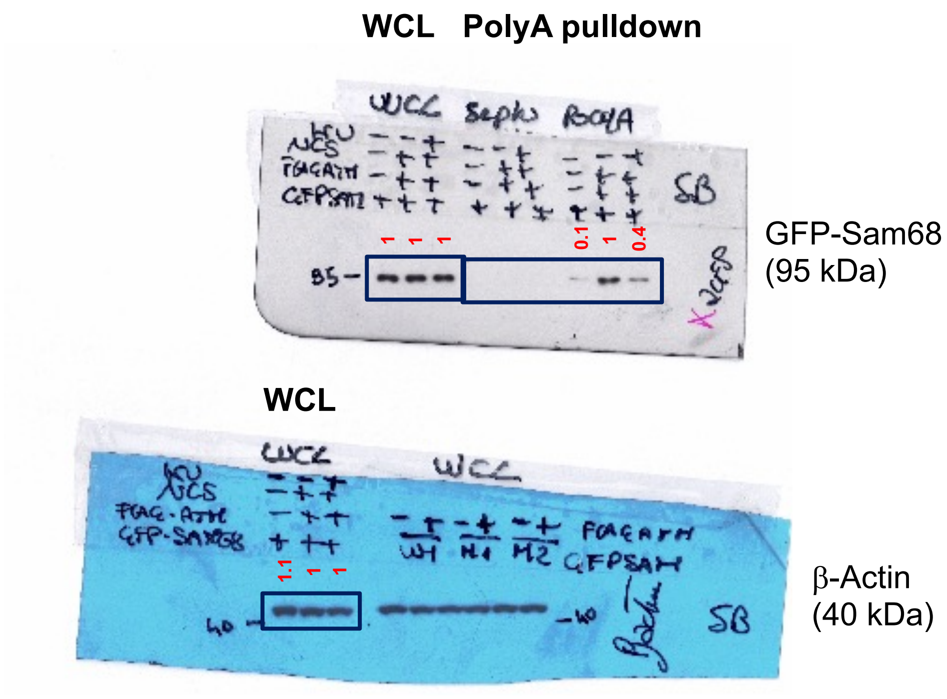


**Figure S1.** Uncropped Western blot of Figure 5B and normalized densitometry in red.

## Figure 5C

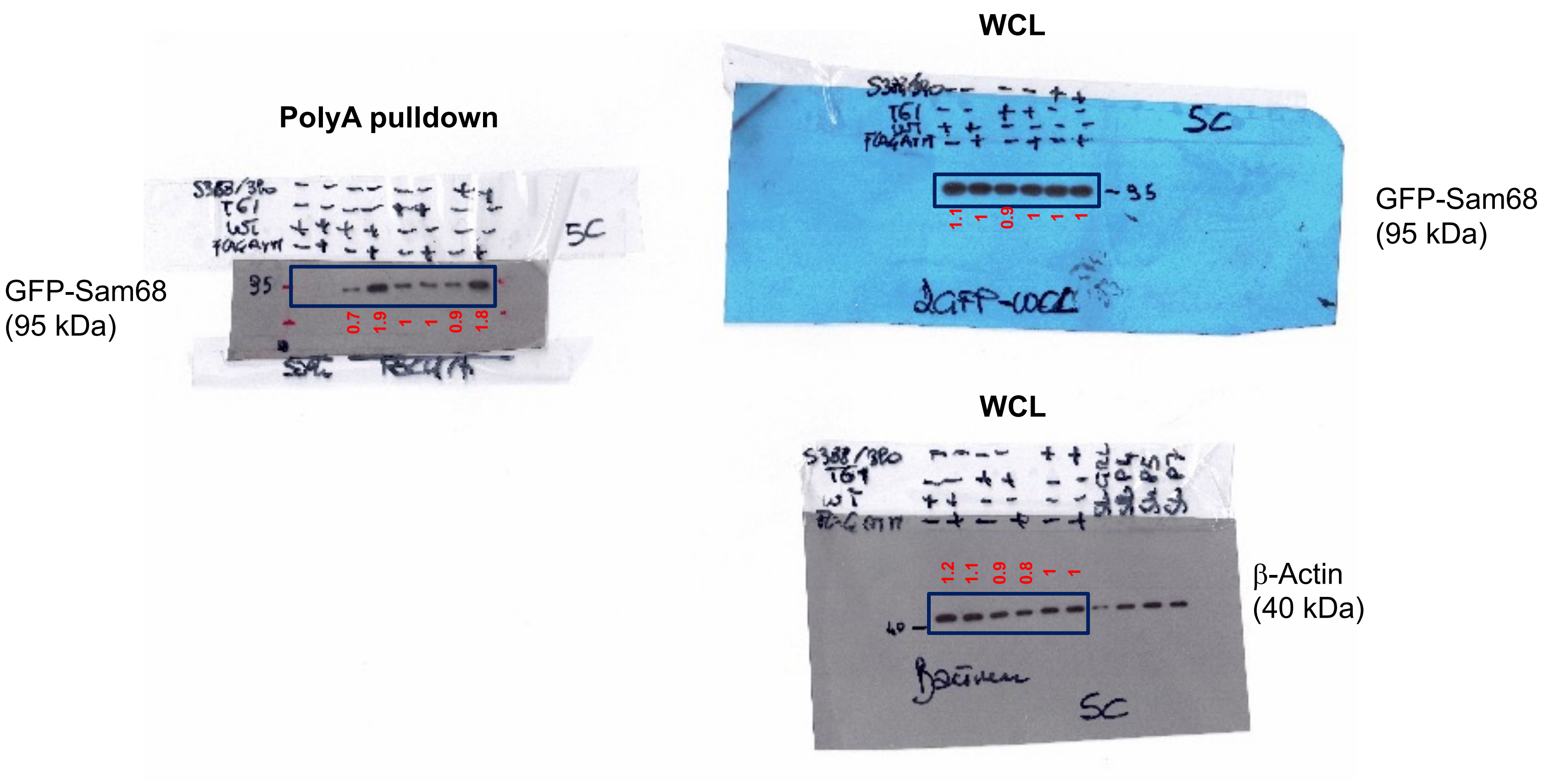


**Figure S1.** Uncropped Western blot of Figure 5C and normalized densitometry in red.

# Figure 6C

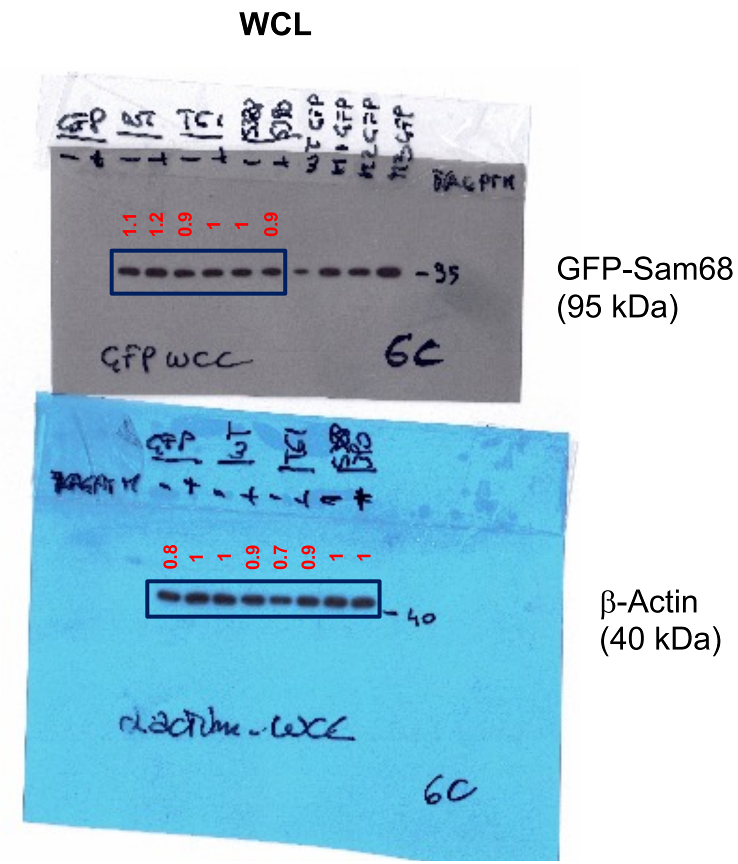


**Figure S1.** Uncropped Western blot of Figure 6C and normalized densitometry in red.

Stagni et al. Supplementary Figure S2

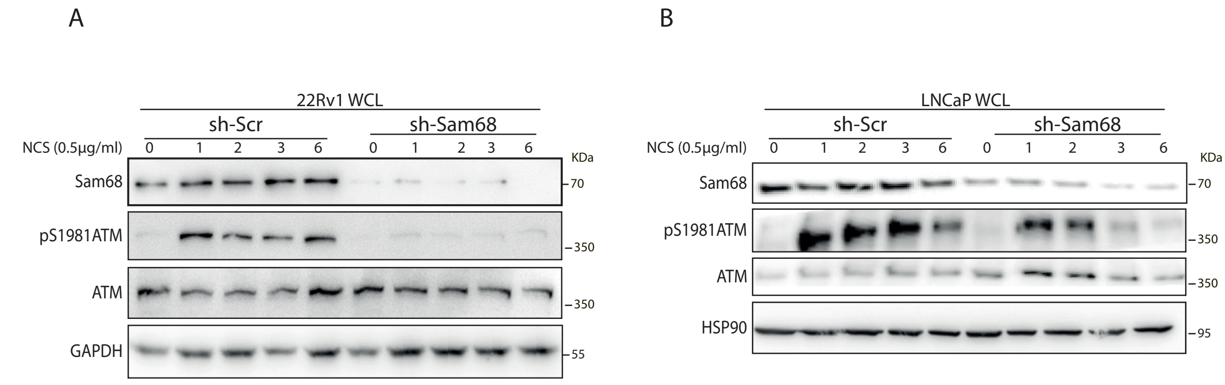


**Figure S2.** Sam68 induces DDR pathway activity. Relative to Figure 1. (A,B) 22Rv1 (**A**) and LNCaP (**B**) cells were silenced (sh-Sam68) or not (sh-Scr) for Sam68 and treated or not with NCS (500 ng/ml) for 1,2,3 or 6 hours. Proteins were extracted and analyzed by Western Blot analysis. (**A**) Representative Western blot of total protein extracts (WCL) were immunoblotted for the indicated antibodies. GAPDH and HSP90 were evaluated as loading controls of total protein extracts.

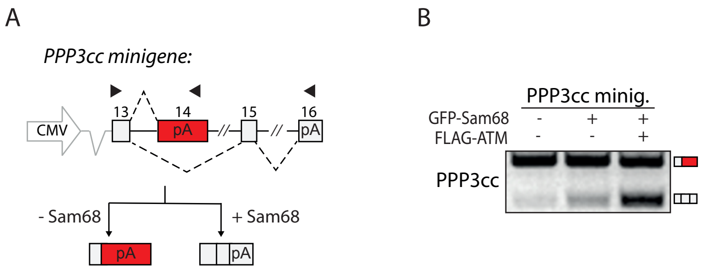


**Figure S3.** ATM modulates APA activity of Sam68. Relative to Figure 5. (**A**) Schematic representation of PPP3CC minigene. The exon 14 in red is the premature last exon, while the exon 16 contains the canonic dpA; Black arrows indicate primers used for the PCR analysis. (**B**) Representative PCR agarose gel of pA selection in PPP3CC minigene system transfected in HEK293T cells in presence, or not, of GFP-Sam68 and FLAG-ATM plasmids.
