# Supplementary material for: DNA Damage Regulates the Functions of the RNA Binding Protein Sam68 through ATM-Dependent Phosphorylation
Source: Cancers (Basel). 2022 Aug 9;14(16):3847. doi: 10.3390/cancers14163847 (PMC9405969; doi:10.3390/cancers14163847)
Supplement: Supplementary file 1 [file cancers-14-03847-s001.zip › cancers-1727942-supplementary Table S1.pdf]

Supplementary Table S1.
List of the oligonucleotides used in this study.

	**Forward**	**Reverse**
**PPP3CC**	GGTGTCCACTCCCAGTTCAA	CAGGTCCAATGGGTGAGCAG (EX14)<br>GGTCCTTCTTGACGCCTGTG (EX16)
**SETMAR dpA**	AGCATTACATCTGAGAAGT	TGTGCAACATGCGGTCGGGC
**SETMAR IPA**	TGAGAAGGAACCCAGCATGT	TTCCACTATAGGGAGGGGCA
**PRKACB IPA**	AGTACCAGATTATCTCTCCCCATGT	TGGCAAAAATTCCACTTTCATGACT
**PRKACB S dpA**	GTGAAAGCACCTTGTAAACTGTAAC	CCTAGCTACAGTAGTGCATAGGAA
**CD44 minigene**	CCTGGTGTGTGGGGAGCGT	CCACCCAGCTCCAGTTGTGCCA
